# Circadian Biomarkers in Humans: Methodological Insights into the Detection of Melatonin and Cortisol

**DOI:** 10.3390/biom15071006

**Published:** 2025-07-14

**Authors:** Cene Skubic, Urša Zevnik, Katarina Nahtigal, Leja Dolenc Grošelj, Damjana Rozman

**Affiliations:** 1 Center for Functional Genomics and Bio-Chips, Institute of Biochemistry and Molecular Genetics, Faculty of Medicine, University of Ljubljana, Zaloška cesta 4, 1000 Ljubljana, Slovenia; cene.skubic@mf.uni-lj.si (C.S.); katarina.nahtigal@mf.uni-lj.si (K.N.); 2 Institute of Clinical Neurophysiology, University Medical Centre Ljubljana, 1000 Ljubljana, Slovenia; leja.dolenc@kclj.si; 3 Department of Neurology, Faculty of Medicine, University of Ljubljana, 1000 Ljubljana, Slovenia

**Keywords:** melatonin, DLMO, cortisol, LC–MS/MS, ELISA, circadian rhythms

## Abstract

Circadian rhythms are intrinsic, with roughly 24 h oscillations that coordinate many physiological functions and are increasingly recognized as key determinants of human health. When these rhythms become misaligned, there is an increased risk for neurodegenerative and psychiatric disorders, metabolic syndrome, sleep disturbances, and even certain cancers. The hormones, melatonin that rises in the evening and cortisol that peaks shortly after awakening, represent crucial biochemical markers of the circadian phase. This review systematically evaluates contemporary techniques for quantifying melatonin and cortisol, comparing biological matrices (blood, saliva, urine) alongside analytical platforms. Special focus is placed on two clinically informative markers: Dim Light Melatonin Onset (DLMO) and the Cortisol Awakening Response (CAR). We compared immunoassays with liquid chromatography tandem mass spectrometry (LC MS/MS), highlighting differences in sensitivity, specificity, and laboratory feasibility. Potential confounders, including ambient light, body posture, and exact sampling times—are discussed in detail, to show the capacity of providing the most reliable results. By emphasizing the need for standardized protocols and controlled sampling conditions, this review provides essential guidance for researchers and clinicians aiming to assess the circadian biomarkers melatonin and cortisol with precision since they can be used in clinical practice as diagnostic and prognostic tools for assessing numerous pathologies.

## 1. Introduction

Circadian rhythms (*circa diem* = about a day) are endogenous, near-24 h cycles that orchestrate a wide range of physiological processes in humans, including the sleep–wake cycle, hormone secretion, metabolism, and behavior [1]. These rhythms persist even in the absence of external cues, reflecting the activity of an internal biological clock [2]. In humans, the primary environmental synchronizer is light, which entrains the suprachiasmatic nucleus (SCN), the master pacemaker located in the hypothalamus. Light signals modulate the expression of core clock genes, including the transcriptional activators *CLOCK* and *BMAL1* (ARNTL1), and the repressor genes *PER* and *CRY*, through tightly regulated transcription–translation feedback loops [3]. This molecular mechanism drives rhythmic outputs from the SCN that are propagated to peripheral clocks via neural, hormonal, and behavioral pathways, prominently involving the hormones melatonin and cortisol [4].

Approximately 80% of protein-coding genes exhibit circadian expression patterns, underscoring the system’s broad physiological impact. Disruption of circadian rhythms has been implicated in a wide spectrum of disorders, including neurodegenerative diseases [5], cancer [6,7], diabetes [8], cardiovascular conditions [9], psychiatric illnesses [10], and sleep disorders such as insomnia, delayed and advance sleep–wake phase disorders [11], and sleep apnea [12,13,14]. In addition the hepatic drug metabolism system is long known to be under circadian control [15]. Thus, the circadian regulation of drug targets has revealed new opportunities for chronotherapy, where timing of medication can improve efficacy and reduce side effects [16,17]. Despite this progress, many mechanistic aspects of circadian disruption in disease remain poorly understood.

**Figure 1:**
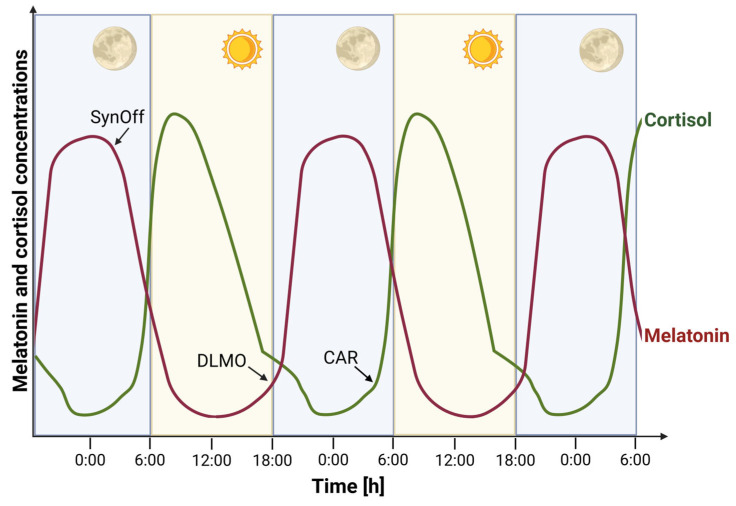
Circadian variation in melatonin and cortisol concentrations over a 48 h period. The figure illustrates the rhythmic secretion of melatonin (red line) and cortisol (green line) aligned with the light–dark cycle. Melatonin levels begin to rise in the evening, peak during the night, and sharply decline in the early morning, marking the synthesis offset (SynOff). The dim light melatonin onset (DLMO) indicates the start of melatonin secretion under dim-light conditions. Cortisol follows a diurnal rhythm, characterized by a sharp increase upon waking—known as the cortisol awakening response (CAR)—followed by a gradual decline throughout the day. Created in BioRender. Nahtigal, K. (2025) https://BioRender.com/6coqad3.

Because direct measurement of SCN activity is not feasible in humans, peripheral biomarkers such as melatonin and cortisol are commonly used as proxies for circadian phase. Melatonin, secreted by the pineal gland in response to darkness, signals the onset of the biological night (Figure 1). Its rise under dim light conditions known as the dim Light Melatonin Onset (DLMO). DLMO is considered the most reliable marker of internal circadian timing [18]. Cortisol, a glucocorticoid hormone produced by the adrenal cortex, shows a characteristic diurnal rhythm with a morning peak. The Cortisol Awakening Response (CAR), a sharp rise in cortisol levels within 30 to 45 min after waking, serves as an index of hypothalamic–pituitary–adrenal (HPA) axis activity and is influenced by circadian timing, sleep quality, and psychological stress [19]. Reliable quantification of these hormones is essential for both research and clinical applications. Saliva sampling has gained popularity due to its non-invasive nature and suitability for repeated, ambulatory measurements. However, low hormone concentrations in saliva challenge analytical sensitivity. Serum, while offering higher analyte levels and better reliability, is more invasive and logistically demanding. Traditionally, immunoassays have been used for hormone measurement, but they suffer from cross-reactivity and limited specificity which is especially problematic for low-abundance analytes like melatonin. Liquid chromatography–tandem mass spectrometry (LC–MS/MS) has emerged as a superior alternative, offering enhanced specificity, sensitivity, and reproducibility for salivary and serum hormone [20,21].

This review critically examines current methodologies for measuring melatonin and cortisol in humans, with a focus on their application in circadian rhythm assessment. By comparing traditional and advanced techniques and incorporating insights from our own research, we aim to clarify best practices and address analytical challenges. The combined use of DLMO and CAR offers a robust framework for evaluating circadian phase and stress reactivity. Reliable quantification of these biomarkers not only supports research but also enhances clinical diagnostics in the emerging field of circadian medicine.

## 2. Melatonin and Cortisol as Endocrine Markers of Circadian Rhythms

### 2.1. Melatonin

Melatonin is a hormone produced by the pineal gland that promotes sleep. Its secretion follows a daily rhythm, with levels reaching nadir during the day and peaking in the early part of the night. The onset of melatonin production-DLMO-typically occurs 2–3 h before sleep [22] and is widely used as a marker of the phase of the endogenous circadian system.

To assess DLMO, it is usually not necessary to monitor the full 24 h melatonin profile. Instead, a 4–6 h sampling window, from 5 h before to 1 h after habitual bedtime is sufficient [23]. The timing of sampling depends on the suspected circadian rhythm disorder (e.g., advanced sleep phase syndrome) and the age of the patient [24,25]. In some cases, such as in blind individuals [26], those with irregular sleep–wake cycles, or patients with alcoholism [27], predicting DLMO is challenging, and an extended sampling period may be necessary to ensure accurate assessment.

Several methods have been proposed to determine DLMO from partial melatonin profiles. The most commonly used is a fixed threshold method, where DLMO is defined as the time when interpolated melatonin concentrations reach 10 pg/mL in serum or 3–4 pg/mL in saliva. Thresholds vary between studies depending on assay sensitivity and the wide inter-individual variation in melatonin production. For low producers—individuals with consistently low melatonin levels—a lower threshold such as 2 pg/mL in plasma may be applied [21,22].

An alternative approach uses a dynamic threshold, defined as the time when melatonin levels exceed two standard deviations above the mean of three or more baseline (pre-rise) values [21,28]. While this method avoids issues with low producers, it becomes unreliable if baseline values are too few (fewer than three) or inconsistent (e.g., due to steep changes in the curve) [29,30].

A study comparing a variable threshold with a fixed 3 pg/mL threshold in saliva samples from 122 individuals found that the variable method produced the DLMO estimates by 22–24 min earlier, but closer to physiological onset in 76% of cases [31]. A similar study favored the fixed threshold, arguing that the variable method produced inaccurate phase estimates due to unstable baselines and thresholds falling below the assay’s functional sensitivity [30].

For a more objective and automated assessment, Danilenko et al. [29] developed the “hockey-stick” algorithm, which estimates the point of change from baseline to rise in melatonin levels for both salivary and plasma samples. When compared with expert visual assessments, the algorithm showed better agreement than either fixed or dynamic threshold methods.

Besides DLMO, melatonin synthesis offset (SynOff)—the time melatonin production ceases—can also be used as a circadian marker [22,32]. Unlike DLMO, SynOff is not defined by a threshold and is unaffected by amplitude. However, it requires frequent sampling across the night, which can be impractical.

Each DLMO estimation method has its strengths and limitations, and no universal standard has been established so far. The choice of method should consider the variability in sample profiles and overall melatonin levels. Wherever possible, results should be confirmed by visual inspection and, if necessary, recalculated using alternative thresholds.

Although melatonin is commonly associated with sleep, it affects nearly every organ and cell in the body [33]. Its functions include free radical scavenging, antioxidant activity, regulation of bone formation, reproduction, cardiovascular and immune function, body mass regulation, and even cancer prevention [34]. For instance, increased rates of breast and colorectal cancer among night shift workers suggest a possible link between reduced melatonin secretion and nocturnal light exposure [35,36]. Suppressed nighttime melatonin has also been reported in Alzheimer’s disease [37,38], autism spectrum disorder [39], and has been discussed as an adjuvant therapy in cardiovascular disorders [40].

Given its broad physiological roles, melatonin measurement will remain a crucial tool in future biomedical research, across a wide range of clinical disciplines.

### 2.2. Cortisol

It is widely known as the body’s stress hormone, cortisol is one of the major glucocorticoids secreted by the adrenal cortex. Its circadian rhythm is roughly opposite to that of melatonin (Figure 1), as cortisol levels peak early in the morning and reach their nadir around midnight. The onset of cortisol’s quiescent phase has been shown to be phase-locked to melatonin onset, making it a potential marker for assessing the phase of the SCN [41].

However, when comparing the two markers, melatonin-based methods offer greater precision. Klerman et al. [42] found that melatonin allows for SCN phase determination with a standard deviation (SD) of 14 to 21 min, whereas cortisol-based methods yielded a less precise SD of about 40 min. Still, melatonin assessment is not always reliable. Factors such as sleep deprivation [43], melatonin supplementation, certain antidepressants [44], and contraceptives [45] can artificially elevate melatonin levels, while non-steroid anti-inflammatory drugs [18] and some beta-blockers [46] may suppress it.

Although cortisol is not a robust marker, it remains a valid alternative to melatonin, and a useful proxy for assessing rhythmicity of the hypothalamic–pituitary–adrenal axis. Given that LC–MS/MS enables simultaneous analysis of both cortisol and melatonin without additional cost or time, measuring both hormones provides a more comprehensive insight into circadian interactions.

Another distinctive feature of cortisol secretion is the cortisol awakening response (CAR), a rapid increase in cortisol levels within 20–30 min of waking [47]. This response is superimposed on the circadian rise in early morning cortisol and is regulated by different mechanisms than the rest of the diurnal cortisol cycle [48]. Evidence suggests that CAR intensity may be influenced by psychosocial factors, such as stress and burnout, although the exact relationships remain unclear [49]. Like the circadian cortisol rhythm, CAR is typically assessed using salivary samples collected immediately upon waking and at set intervals over the following hour.

Precise cortisol measurement is also essential in clinical diagnostics, particularly for conditions involving adrenal insufficiency or excess cortisol production, where a loss of normal rhythmicity is a hallmark feature [50]. Beyond its circadian roles, cortisol plays a crucial role in energy metabolism, cardiovascular function, respiratory regulation, blood flow redistribution, and immune modulation [47]. Dysregulation of cortisol rhythms has been implicated in neurodegeneration [51], increased cardiovascular risk [52], and sleep disturbances, particularly a higher proportion of wake after sleep onset (WASO) [53]. Thus, cortisol assessment holds value well beyond the field of circadian research. While recent studies suggest that higher CAR in healthy adults may reflect greater life stress and lower psychopathology, pointing to the HPA axis as a marker of resilience [54], reduced CAR in individuals with personality disorders has been associated with nonadaptive thinking patterns such as external attribution bias, demonstrating its relevance of both resilience and vulnerability [55].

### 2.3. Cofounding Factors

Although both hormones are recognized as robust markers of central circadian phase, their measurement can be influenced by medications, behaviors and environmental conditions, potentially compromising accuracy or rendering the results invalid (see Table 1 for a summary of masking factors).

For example, ambient light not only suppresses melatonin secretion but can also shift the phase of the SCN, thereby affecting both melatonin and cortisol rhythms. Light intensities as low as 6 lux can suppress melatonin levels by up to 50% in highly light-sensitive individuals, highlighting the need to conduct assessments under dim light conditions, typically below 30 lux [76]. While some masking effects, like light exposure are well documented, other influences may be more subtle yet still critical to consider for accurate assessment of circadian hormone profiles.

Studies show that the consumption of melatonin-rich foods, such as sour cherry juice, kiwis, cereals, pulses, nuts, olive oil and vegetables can significantly increase circulating melatonin concentrations and even influence the rhythms of urinary excretion. These results show how important it is to control food intake when quantifying melatonin in different biological matrices, as melatonin from food can distort the results of analyses [77,78].

### 2.4. Alternative Circadian Biomarkers

In addition to melatonin and cortisol, several other biomarkers provide valuable insights into circadian timing. Core body temperature [79] shows strong circadian rhythmicity, with higher amplitude linked to greater metabolite oscillations. Skin temperature rhythms have been shown to differ in individuals with mood disorders, offering a non-invasive peripheral marker [80]. Locomotor activity, often measured via actigraphy or wearables, reflects rest-activity cycles and is widely used in field studies [81]. At the molecular level, circadian transcriptomics and proteomics reveal rhythmic gene and protein expression patterns in both central and peripheral tissues [82]. Recent studies also highlight the role of brain circadian clocks beyond the SCN in regulating behavioral rhythms [83]. These markers complement hormonal measures and offer expanded tools for circadian assessment.

## 3. Sampling Strategies in Different Matrices

### 3.1. Serum or Plasma

Chronobiological research has traditionally been conducted in controlled clinical settings. Participants typically receive cannulas indwelling, and blood samples are collected at regular intervals over several hours. This approach offers several advantages: it provides access to higher concentrations of metabolites, allows for sampling during sleep, and enables the concurrent analysis of other relevant biomarkers, including plasma metabolites and mRNA. However, the method also has notable drawbacks. It is invasive and burdensome for participants and may lead to artificially elevated cortisol levels due to stress induced by hospital visits and repeated blood draws [84]. Moreover, the procedure requires trained medical personnel, immediate sample processing (including overnight), and specialized infrastructure, making it both costly and impractical for large-scale studies.

### 3.2. Saliva

In contrast to blood-based sampling, salivary hormone collection is cost-effective, non-invasive, and can be performed at home. Samples should be stored cold or frozen until delivery. During the collection period, participants are instructed to remain in dim light, refrain from alcohol, caffeine, and toothpaste use, and avoid food intake for at least 30 min prior to sampling to prevent contamination with dietary melatonin. Some protocols also recommend rinsing the mouth with water 10–15 min before collection; to ensure comparability across individuals. However, this timing should be standardized [21,24].

Samples visibly contaminated with blood should be discarded and recollected, as blood contamination may artificially elevate cortisol levels [85]. Although full compliance with these instructions cannot be guaranteed, studies have validated that self-collected saliva samples can reliably determine DLMO [31,86]. To enhance reliability, objective monitoring of environmental illumination, for example, through wearable devices, is strongly recommended [25].

**Figure 2:**
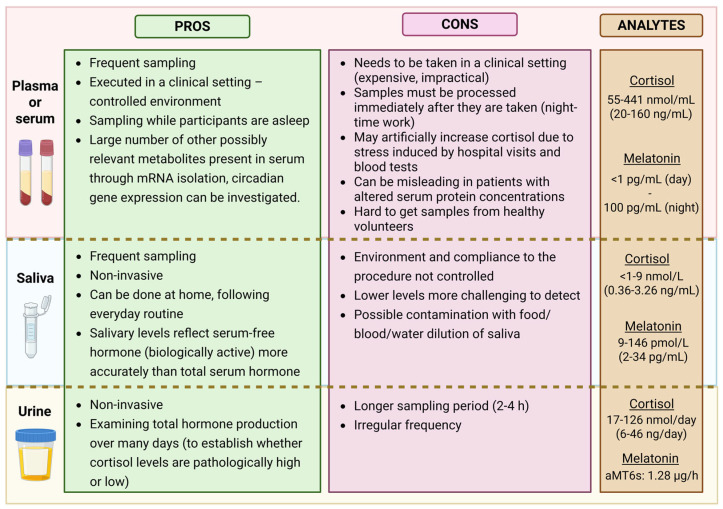
Comparison of different sampling options with pros and cons for each biological sample. The concentration range of analytes is also shown. Created in BioRender. Nahtigal, K. (2025) https://BioRender.com/m3x4zx7.

When comparing plasma and saliva (see Figure 2), it is important to consider that approximately 70% of melatonin and 95% of cortisol in plasma are protein-bound. Only the unbound (free) fraction is biologically active and capable of diffusing into other fluids such as saliva. Salivary melatonin concentrations are typically 24–33% of those found in plasma [87], while salivary cortisol ranges between 50 and 70% of the free cortisol in serum [88].

Numerous studies have shown that salivary hormone levels reflect free serum concentrations more accurately than total serum levels over a 24 h period, particularly in individuals with altered protein-binding capacities—such as those with liver disease, high estrogen levels (e.g., pregnancy or contraceptive use) [89]. Therefore, saliva is a suitable medium for assessing circadian rhythms [84,87]. However, for the evaluation of pineal or adrenal gland function, salivary concentrations should not be extrapolated to total plasma levels [21].

On the downside, lower melatonin and cortisol concentrations in saliva may pose technical challenges for certain assays—especially when hormone production is at its nadir (i.e., during the light phase for melatonin or during the night for cortisol). Titman et al. [84] reported that in 19% of afternoon samples (after 13:00) collected from children, salivary cortisol was undetectable. Likewise, in a study by Crowley et al. [30], almost half of the 66 adolescents studied had 2SD thresholds lower than the functional assay sensitivity (<0.9 pg/mL), rendering accurate analysis difficult. To address this, careful selection of analytical methods is essential.

Measuring salivary melatonin accurately is particularly demanding due to its low concentration. According to updated guidelines by Kennaway [21], only assays with a limit of quantification (LOQ) below 3 pg/mL that can reliably detect pre-DLMO levels are considered appropriate. Both highly sensitive immunoassays and LC–MS methods, if properly validated, are suitable for assessing circadian timing via saliva DLMO. This technique is especially useful for evaluating circadian responses to jet lag, light exposure (timing and wavelength), disease, or medications.

It is important to note that single-timepoint saliva melatonin measurements have no meaningful physiological value. Furthermore, salivary melatonin should not be used to estimate total melatonin production, due to significant individual variability in plasma protein binding across different ages and health states. Assays that consistently report daytime saliva melatonin levels above 3 pg/mL should be avoided, as they likely indicate falsely elevated values, undermining the reliability of circadian research outcomes.

### 3.3. Urine

A small fraction—approximately 2% of unbound plasma cortisol—is excreted in the urine and can be detected as urine-free cortisol [89]. In contrast, melatonin is assessed via its primary urinary metabolite, 6-sulfatoxymelatonin (aMT6s).

Urine sampling, like salivary sampling, is non-invasive, can be performed at home, and does not require immediate sample processing, making it a practical alternative in many research and clinical contexts. However, unlike blood or saliva sampling, it does not permit high-frequency collection or real-time tracking of hormone fluctuations. Instead, it serves as a proxy for total hormone production over an extended period, offering valuable insight into cumulative endocrine output.

This makes urinary hormone measurements particularly useful in medical diagnostics and research. For example, 24 h urine-free cortisol is commonly used to screen for Cushing’s syndrome, a condition of cortisol excess. Studies have shown that urinary cortisol levels correlate well with mean serum-free cortisol, except in cases of severe renal impairment [89]. Similarly, morning aMT6s concentrations have been found to correlate with sleep quality and are typically reduced in individuals with sleep disorders [90].

### 3.4. Alternative Sampling Options

While blood, saliva, and urine remain the primary sources for measuring cortisol and melatonin, alternative biological matrices are increasingly being explored. A review of the literature reveals that most detection methods prioritize cortisol over melatonin, likely due to the growing interest in stress monitoring. Fingertip sweat sensors [91,92] and soft contact lenses [93] have utilized selective binding approaches, either through antibodies or cortisol-imprinted electropolymerized coatings, combined with electrochemical sensing to monitor endocrine responses to stress and circadian fluctuations.

Another promising technology is the U-RHYTHM device, which is inserted subcutaneously and uses microdialysis to collect samples continuously. After retrieval, the collected dialysate can be analyzed for various metabolites and steroids, including tissue-free melatonin and cortisol [94]. This approach enables the observation of both diurnal and ultradian rhythms, as well as their interaction and potential modulation by environmental and behavioral factors.

Although not relevant for assessing circadian rhythms, hair cortisol measurement holds significant clinical value. The procedure involves segmenting hair into sections that correspond to defined time periods, extracting cortisol, and quantifying it using standard analytical techniques [95]. Longer hair samples can reflect cortisol production over several months to years [96], providing insights into long-term exposure to chronic stress, as well as the temporal progression of adrenal insufficiency or Cushing’s syndrome [95].

These alternative biomarkers, adaptable to both free-living and clinical settings, offer promising avenues for self-monitoring, research, and clinical diagnostics. However, further validation studies are essential before their widespread adoption.

## 4. Analytical Techniques for Melatonin and Cortisol Quantification

Measuring melatonin and cortisol in biological samples presents two major methodological challenges: their extremely low concentrations at circadian nadirs (below 1 pg/mL for melatonin and below 1.0 ng/mL for cortisol in saliva), and the complexity of biological matrices, which often contain structurally similar interfering compounds. Although immunoassays currently dominate in routine testing, liquid chromatography-mass spectrometry (LC–MS) methods are gaining attraction—not only in research settings but increasingly in clinical laboratories—due to their superior specificity and sensitivity.

### 4.1. Immunoassays (IA)

The exceptional specificity of antibodies, coupled with our ability to generate them against virtually any (macro)molecule, underpins the widespread applicability of immunoassays. In these assays, antibodies are designed to selectively bind target analytes. The resulting binding is detected via an indicator molecule and quantified by comparison to a calibration curve. Immunoassays are generally classified into competitive and non-competitive. In competitive assays, the analyte in the sample competes with a labeled analog for binding to the antibody, and the signal is inversely proportional to the analyte concentration. In contrast, non-competitive assays use labeled antibodies that bind directly to the antigen; the bound complexes are retained while unbound antibodies are washed away, and the signal is directly proportional to the analyte concentration.

For melatonin and cortisol measurements, the label may be: a radioisotope (radioimmunoassay, RIA), an enzyme that catalyzes a detectable product (enzyme-linked immunosorbent assay, ELISA), or a luminescent molecule (chemiluminescent or electrochemiluminescent immunoassays, CLIA or ECLIA).

The broad use of immunoassays is due to their commercial availability, affordability, minimal equipment requirements, ease of use, suitability for automation, and ability to process multiple samples simultaneously. However, one of their major limitations lies in the difficulty of producing antibodies with complete specificity. Cross-reactivity with structurally similar molecules can lead to artificially elevated readings. For instance, melatonin immunoassays may also detect serotonin, N-acetylserotonin, N-acetyl-N-formyl-5-methoxytryptamine, and 5-methoxytryptamine. Similarly, cortisol assays may cross-react with other steroids, particularly cortisone, whose concentrations can be up to three times higher than cortisol [89]. This issue is especially pronounced at low analyte concentrations [97].

As a result, and due to differences in assay validation, substantial discrepancies have been observed between various commercial immunoassay kits. A study comparing commonly used immunoassays with a reference LC–MS/MS method across 195 saliva samples representing the full adult cortisol range found the results to be poorly aligned [98]. LC–MS/MS also shows that expected plasma melatonin levels during the light phase are typically 1–3 pg/mL, with salivary melatonin levels representing 30–40% of plasma concentrations. Yet, numerous studies using commercial immunoassay kits have reported melatonin levels exceeding 100 pg/mL during daylight hours, even though the pineal gland does not produce melatonin at that time. ELISA kits, which dominate the melatonin assay market, were found to show even higher daytime melatonin levels and lower sensitivity compared to RIA [99]. However, a recent independent comparison demonstrated that the improved Novolytix ELISA (MLTN-96) yields salivary melatonin concentrations and DLMO estimates closely aligned with the gold-standard RIA (RK-DSM2), suggesting that when rigorously validated, ELISAs can offer a reliable and practical alternative for circadian research [100].

In terms of sensitivity, a critical review of 21 commercial melatonin immunoassay kits concluded that many are unsuitable for accurately measuring daytime levels or determining DLMO due to insufficient sensitivity [21,99]. Similarly, salivary cortisol was undetectable in 19% of post-13:00 samples in children [84] and in 30% of samples from healthy adults [20], highlighting a comparable issue in cortisol measurement.

### 4.2. Liquid Chromatography–Tandem Mass Spectrometry

LC–MS/MS combines the high analytical sensitivity of mass spectrometry with the powerful separation capabilities of chromatography, offering superior specificity, sensitivity, and linearity compared to immunoassays. One of its key advantages lies in the ability to distinguish between structurally similar compounds, thereby eliminating the issue of cross-reactivity. Endogenous interferences are minimal, but may include salts, especially in urine samples, and co-eluting substances that can reduce ionization efficiency [89]. To address these challenges, a variety of sample pre-treatment and extraction techniques are employed, depending on the biological matrix and analyte of interest. The studies summarized in Table 2 primarily utilize:

- Protein precipitation (PPT) [97,101,102];
- Liquid–liquid extraction (LLE) [20,103,104];
- Solid-phase extraction (SPE) [101,105,106,107].

While PPT is the simplest and fastest method, LLE and especially SPE offer greater purification but are more technically demanding, time-consuming, and costly. These limitations make them less suitable for high-throughput clinical settings [102].

**Table 2:** Comparison between LC–MS/MS and immunoassays for measuring melatonin or cortisol.

Reference	What Did They Compare?	Conclusion
[20]	LC–MS/MS vs. ELISA (melatonin) and ECLIA (cortisol) in 121 salivary samples from healthy subjects.	Strong correlation (r = 0.910 for melatonin, r = 0.955 for cortisol), but IA showed significant positive bias; 30% of cortisol samples fell below ECLIA LLOQ; LC–MS/MS required less sample volume.
[107]	Two LC–MS/MS methods and RIA (Bühlmann) on salivary melatonin from 39 patients.	LC–MS/MS methods showed strong agreement (r = 0.99); RIA had greater variance (r = 0.74, mean bias −11.7%). LC–MS/MS was superior in precision and trueness.
[106]	ELISA vs. LC–MS/MS on 35 salivary melatonin samples.	Good agreement in low range; above 30 pmol/L, ELISA underestimates. Mean bias 7.9 pmol/L. Calibration difference excluded as source.
[108]	Two CLIA (ADVIA, LIAISON) vs. LC–MS/MS on 24 h urinary cortisol in 174 patients.	Strong correlation overall; discrepancies at high cortisol (>500 µg/mL), with IA reading 2–9× higher than LC–MS/MS.
[101]	IA vs. LC–MS/MS on 2703 salivary cortisol samples from children	IA measured values ~2.39× higher. Cross-reactivity with cortisone affected results <5 nmol/L. Over 50% of samples were in this range.
[97]	ECLIA vs. LC–MS/MS on 324 late-night salivary cortisol samples.	High correlation (r² = 0.892), but high bias at low concentrations. In total, 68.8% of reference samples were under ECLIA detection limit.
[102]	Routine immunoassays and LC–MS/MS vs. cRMP in serum cortisol across multiple cohorts.	LC–MS/MS closely matched cRMP. IA results varied by cohort: underestimation in pregnancy, overestimation in metyrapone/prednisolone groups.
[105]	ECLIA (Cortisol I and II) vs. LC–MS/MS on stimulated serum cortisol.	Cortisol II showed small bias (~4% lower); Cortisol I overestimated by ~36%. LC–MS/MS supports lower diagnostic cutoffs.
[103]	ELISA, RIA, and LC–MS/MS in inter-laboratory salivary melatonin and cortisol comparison.	High inter-lab variability. ELISA overestimated melatonin (6.90 vs. 0.278 pmol/L). LC–MS/MS results varied among labs.
[104]	EIA vs. LC–MS/MS on salivary cortisol (bedtime and morning) in 53 subjects.	Excellent correlation (r² = 0.97); LC–MS/MS consistently yielded lower values, likely due to reduced cross-reactivity.

A significant advantage of LC–MS/MS is its ability to simultaneously quantify multiple analytes. Several validated protocols allow for the concurrent measurement of salivary [20,103] and plasma [106] melatonin and cortisol. However, unlike immunoassays, LC–MS/MS lacks parallel processing capacity, making it inherently slower. Furthermore, it requires costly instrumentation and highly trained personnel, which limits its widespread use in clinical and routine laboratory settings despite its analytical superiority.

Gas chromatography-mass spectrometry (GC–MS) is another highly sensitive and accurate technique capable of detecting low levels of cortisol and melatonin. However, its application is constrained by the need to derivatize non-volatile compounds before analysis [102], resulting in a labor-intensive and lower-throughput workflow. Consequently, GC–MS remains largely restricted to specialized research and reference laboratories [89].

### 4.3. Comparison of Immunoassay and LC–MS/MS Methods

**Figure 3:**
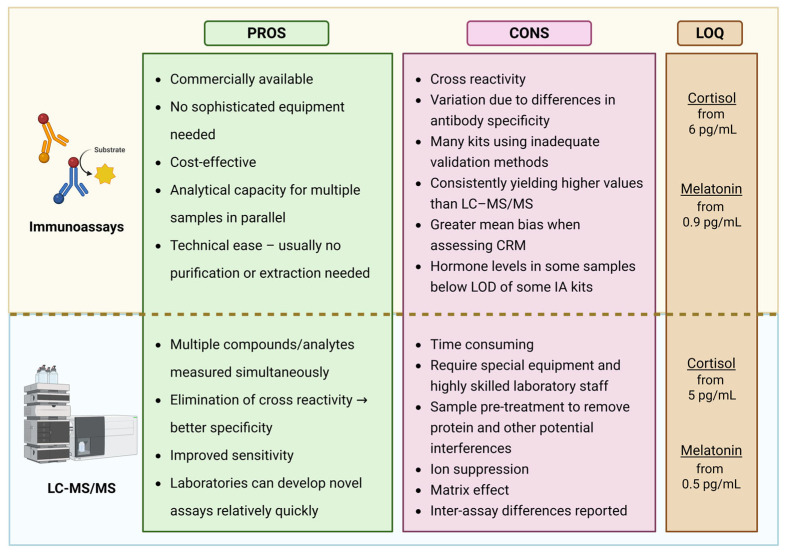
A comparison between immune assays and liquid chromatography with tandem mass spectrometry (LC–MS/MS). Pros and cons for each methodology is listed, together with level of quantification (LOQ). Created in BioRender. Nahtigal, K. (2025) https://BioRender.com/cus4eey.

An analysis of research comparing immunoassays and LC–MS/MS (Figure 3) reveals that most studies focus on cortisol quantification, primarily to establish diagnostic cut-off values for cortisol excess or deficiency. To provide context and highlight broader trends, some earlier melatonin studies are also included.

The literature agrees that LC–MS/MS and immunoassays generally show good correlation, indicating that both methods are capable of tracking circadian rhythms. However, a consistent finding across studies is that immunoassays tend to yield higher analyte concentrations, regardless of the fluid type or whether cortisol or melatonin is being measured. This discrepancy is most often attributed to variations in assay validation and to antibody cross-reactivity with structurally similar compounds. Although sample purification via column chromatography or solvent extraction can improve specificity, most commercially available immunoassay kits now rely on direct measurement without any pre-treatment, making them more susceptible to cross-reactivity.

In melatonin studies, substantial mean biases have been reported: 49% [20], 12% [107], and 1.7 pg/mL [106]. These deviations challenge the reliability of using fixed thresholds, such as the commonly applied 10 pg/mL cut-off for determining DLMO. Similarly, marked variability in cortisol measurements between assays necessitates assay-specific interpretation of results. Diagnostic cut-offs for adrenal insufficiency or hypercortisolism must be individually validated in each laboratory to ensure diagnostic accuracy and avoid misclassification.

While correlations between immunoassays and LC–MS/MS may be good on average, their ratio is not constant across the full measurement range. Notable discrepancies occur at higher concentrations. For example, ELISA was found to underestimate salivary melatonin levels above 30 pmol/L (7 pg/mL) compared to LC–MS/MS [106]. In urinary cortisol measurements, concentrations above 500 µg/mL showed similar divergence [108]. Conversely, at low cortisol levels (below 5 nmol/L: 1.8 ng/mL) the cross-reactivity with cortisone increasingly skews immunoassay readings [101]. This is particularly problematic in saliva, where cortisone levels may be several-fold higher than cortisol. Even small degrees of cross-reactivity can result in significant overestimation of cortisol when the interfering compound is present in large excess.

Additionally, in physiological states associated with elevated cortisol-binding globulin (CBG), such as pregnancy, immunoassays tend to underestimate total cortisol at higher concentrations. This may result from incomplete displacement of cortisol from its binding proteins during sample processing. Such limitations can be addressed either by measuring salivary cortisol, which reflects free hormone levels and is less affected by CBG, or by using LC–MS/MS. Notably, Hawley et al. [102] reported only a 1.4% mean bias in LC–MS/MS compared to a candidate reference method.

Another significant limitation of immunoassays is their reduced sensitivity at low hormone levels. Two studies on salivary cortisol found that a large proportion of late-night samples fell below the limit of quantification: 30% in healthy adults [20] and 69% in children [97]. This poses a challenge for circadian studies aiming to characterize 24 h hormone profiles, determine cortisol quiescence periods, or define DLMO using dynamic thresholds. The issue is especially pronounced in pediatric populations, who naturally exhibit lower salivary cortisol and melatonin concentrations.

Despite its superior analytical performance, LC–MS/MS is currently rarely used in routine clinical settings due to significant obstacles. These include high initial costs, the need for specialized infrastructure (e.g., controlled environments, high purity gases) and lower throughput compared to automated immunoassay analyzers. Consumables, certified standards and labor-intensive sample preparation further increase operational costs. Finally, method development, troubleshooting and data interpretation require highly trained analytical chemists—expertise that is often lacking outside of specialized reference laboratories. As a result, many clinical laboratories continue to rely on immunoassays, despite its known disadvantages in terms of specificity and accuracy [109].

## 5. Determination of DLMO and CAR in Pediatric Population with Psychiatric Disorders

In children with conditions such as autism spectrum disorder (ASD), attention-deficit/hyperactivity disorder (ADHD), and epilepsy, circadian rhythm disruptions are prevalent and often linked to sleep disturbances, behavioral dysregulation, and seizure susceptibility. Studies have demonstrated altered melatonin and cortisol secretion profiles in children with ASD, ADHD and epilepsy, supporting the clinical utility in diagnosis and treatment planning.

Therefore, these pediatric populations would benefit greatly from the implementation of a quick and efficient method for the determination of DLMO [110] to inform the timing of pharmacological interventions (e.g., melatonin supplementation, corticosteroids) and behavioral therapies. Different studies [86,111,112] have already shown feasible and accurate results using different self-directed in-home DLMO saliva collection kits for immunoassays or LC–MS/MS, that can serve to assess circadian phase in different populations. These approaches could be very beneficial for children with neurological disorders, because there is no need to visit the clinic for the collection of saliva. In addition, appropriate melatonin profiling is needed to avoid the overuse of antiepileptic drug use by melatonin supplementation when needed [113] or for treating primary sleep disorders and the sleep disorders associated with different neurological conditions [114]. In addition, it should be noted that urine sampling is often easier and better tolerated than saliva sampling in younger children and in children with ASD, making it a practical option also for home melatonin measurements [115].

### Recommendations for Standardized Protocols

No matter which essay is used, standardized protocols are essential to ensure accuracy, reproducibility and comparability across studies and clinical applications. Therefore, we are proposing recommendations for standardized protocols for LC/MS-MS and immunoassay methods in Table 3. To analyze circadian data and obtain key parameters such as period, amplitude, mesor, and phase, bioinformatics tools including CircaCompare, CosinorPy, DiscoRhythm, and CircadiPy can be employed [116,117,118,119], which offer robust statistical frameworks for rhythm detection and comparison across groups. Regardless of the specific tool used, standardized analytical protocols are essential to ensure accuracy, reproducibility, and comparability across studies and clinical applications.

## 6. Conclusions

Accurate quantification of melatonin and cortisol is essential for reliable assessment of circadian rhythms in humans. This review highlights how the choice of measurement strategy influences the utility of these hormones as biomarkers of circadian timing. Melatonin marks the onset of the biological night, typically defined by the DLMO, while cortisol reflects the circadian wake-up signal, known as the cortisol awakening response (CAR).

Different biological matrices—blood, saliva, and urine—each offer distinct advantages and limitations depending on the analyte, sampling frequency, study design, and whether the setting is clinical or non-clinical. In addition, environmental and behavioral factors, sampling timing, and analytical sensitivity must all be carefully considered, as they can significantly influence the measured concentrations.

Two main analytical approaches are used: immunoassays and LC–MS/MS. Immunoassays are widely accessible, cost-effective, and operationally simple, making them suitable for many clinical and research contexts. However, they are prone to cross-reactivity and variability across commercial kits, which can compromise specificity and accuracy. In contrast, LC–MS/MS provides superior analytical performance, with higher specificity, sensitivity, and the ability to distinguish structurally related compounds. These advantages make it the preferred method in circadian and endocrine research, despite its higher cost, technical complexity, and limited accessibility.

Ultimately, the choice between immunoassay and LC–MS/MS should be guided by the specific research objectives, required analytical precision, available resources and the context of application.

## Figures and Tables

**Table 1:** Factors that may influence melatonin and cortisol measurement—masking factors.

Change	Melatonin	Cortisol
**Increase** **↑**	**Major:** Contraceptives [45,56], **Minor:** Sleep deprivation [43,57], certain antidepressants [44], standing position versus sitting [58,59].	**Major:** Stress [60], morning light (induces an immediate, greater than 50% elevation of cortisol levels—even after a sleepless night) [61], awakening [60], exercise [62], aging (also shifts cycle), contamination of saliva samples with blood [63], oral contraceptives (women treated with the OCP displayed a 1.7–2.2-fold increase in total plasma cortisol levels) [64]. **Minor:** high protein meals [60] and smoking before saliva collection [63].
**Decrease** **↓**	**Major:** Light (with light at the blue end of the spectrum having the biggest impact) [65,66,67] and certain beta blockers [46] **Minor:** Nonsteroidal anti-inflammatory drugs [18], nocturnal physical activity [68], caffeine (consumed a few hours before measurement) [69], saliva collection with cotton swabs compared with that from passive saliva collection [70], possibly reduced melatonin secretion in the luteal phase in women [71].	**Minor:** Possibly reduced amplitude of cortisol in the luteal phase in women [71].
**Masking** **↑ and ↓**	**Major:** Ethnicity, ancestry and genetics: Caucasian participants were found to have higher daily melatonin levels than Asians [72], African Americans excreted less 6-sulphatoxymelatonin compared to European Americans [73]. Nevertheless, DLMO was not found to vary between races [45].	**Major:** Ethnicity, ancestry: Africans and Hispanics/Latinos have flatter diurnal cortisol slopes [74]. **Minor:** In patients with type 2 diabetes, morning serum cortisol was shown to depend on morning fasting glycemia, while salivary cortisol did not [75].

**Table 3:** Recommendations for standardized protocols for LC–MS/MS and immunoassay methods.

LC–MS/MS	Immunoassay
**Sample collection**	**Assay Selection**
Clearly define time of sample collection relative to expected DLMO (e.g., every 30–60 min, from 18:00 to 00:00). Clearly define time of sample collection relative to expected CAR (immediately upon waking and every additional 15–45 min). Store samples at −80 °C immediately after collection. Use appropriate sample collection tools (e.g., salivettes).	Validated commercial kits should be used. Kit sensitivity: <0.5 pg/mL for melatonin, <1 ng/mL for cortisol. Kit should report cross-reactivity profile and should be validated for studied population. Kit should have <10% intra-and inter-assay coefficient of variation.
**Analytical procedure**	**Sample collection**
Use solid-phase extraction or protein precipitation method for interfering compounds removal. Use stable isotope-labeled internal standards (e.g., D4-cortisol, D4-melatonin) for quantification. Reverse-phase C18 columns are commonly used for chromatography. Use multiple reaction monitoring (MRM) with high specificity transitions (e.g., melatonin m/z 233 → 174) in MS parameters. Limit of quantification (LOQ) should be ≤1 pg/mL for melatonin and ≤0.5 ng/mL for cortisol in saliva.	Avoid food and/or drinks 30 min before saliva sampling. Collect samples under dim light for DLMO assessment. Store samples at −20 °C or lower, and analyze within a defined time window (e.g., <3 months if not frozen at −80 °C).
**Quality control**	**Normalization and interpretation**
Prepare calibration curves using matrix-matched calibrators covering the full physiological range. Include low, mid and high concentration controls in every batch of samples.	CAR needs to be normalized to the baseline. DLMO threshold must be defined and consistent (e.g., 3 pg/mL of melatonin in saliva).

## Abbreviations

The following abbreviations are used in this manuscript:

DLMO	Dim light melatonin onset
CAR	Cortisol awakening response
LC–MS/MS	Liquid chromatography–tandem mass spectrometry
MRM	Multiple reaction monitoring
LOQ	Limit of quantification
SCN	The suprachiasmatic nucleus
RIA	Radioimmunoassay
PPT	Protein precipitation
LLE	Liquid–liquid extraction
SPE	Solid-phase extraction
GC–MS	Gas chromatography–mass spectrometry
ASD	Autism spectrum disorder
ADHD	Attention-deficit/hyperactivity disorder
WASO	Wake after sleep onset
